# Lowering the culture temperature corrects collagen abnormalities caused by HSP47 gene knockout

**DOI:** 10.1038/s41598-019-53962-0

**Published:** 2019-11-22

**Authors:** Kazunori K. Fujii, Yuki Taga, Takayuki Sakai, Shinya Ito, Shunji Hattori, Kazuhiro Nagata, Takaki Koide

**Affiliations:** 1 Department of Chemistry and Biochemistry, School of Advanced Science and Engineering, Waseda University, Shinjuku, Tokyo 169-8555 Japan; 2 Nippi Research Institute of Biomatrix, 520-11 Kuwabara, Toride, Ibaraki 302-0017 Japan; 3 Laboratory of Molecular and Cellular Biology, Faculty of Life Sciences, Kyoto Sangyo University, Kamigamo, Kita-ku, Kyoto 803-8555 Japan

**Keywords:** Endoplasmic reticulum, Chaperones

## Abstract

Heat shock protein 47 (HSP47) is an endoplasmic reticulum (ER)-resident molecular chaperone that specifically recognizes triple helical portions of procollagens. The chaperone function of HSP47 is indispensable in mammals, and *hsp47*-null mice show an embryonic lethal phenotype accompanied by severe abnormalities in collagen-based tissue structures. Two leading hypotheses are currently accepted for the molecular function of HSP47 as a procollagen-specific chaperone. One is facilitation of procollagen folding by stabilizing thermally unstable triple helical folding intermediates, and the other is inhibition of procollagen aggregation or lateral association in the ER. The aim of this study was to elucidate the functional essence of this unique chaperone using fibroblasts established from *hsp47*−/− mouse embryos. When the cells were cultured at 37 °C, various defects in procollagen biosynthesis were observed, such as accumulation in the ER, over-modifications including prolyl hydroxylation, lysyl hydroxylation, and further glycosylation, and unusual secretion of type I collagen homotrimer. All defects were corrected by culturing the cells at a lower temperature of 33 °C. These results indicated that lowering the culture temperature compensated for the loss of HSP47. This study elucidated that HSP47 stabilizes the elongating triple helix of procollagens, which is otherwise unstable at the body temperature of mammals.

## Introduction

Collagens are abundantly expressed extracellular matrix proteins in all multicellular animals, which play crucial roles in maintaining the integrity of tissues and organs. The hallmark of collagens is the triple helical domain consisting of glycine (Gly)-Xaa-Yaa triplet repeats. In mammalian collagens, approximately one third of Xaa and Yaa positions are occupied by proline (Pro) and 4-hydroxyproline (4-Hyp) residues, respectively. Such high content of imino acid residues and post-translational prolyl hydroxylations greatly contribute to stabilizing the triple helical structure^1^. Twenty-eight types of collagen have been identified in humans. Among them, the most abundant fibril-forming types, such as types I, II, and III, have been well studied in terms of their structures, functions, and biosynthetic pathways. Each triple helical collagen molecule is formed by three α-chains with more than 300 uninterrupted repeats of the Gly-Xaa-Yaa triplet.

A triple helical collagen molecule is produced in the extracellular space by enzymatic elimination of N- and C-propeptide domains from a procollagen. Structure formation and post-translational modification processes of procollagens are carried out in the endoplasmic reticulum (ER) lumen. Three pro-α-chains are first trimerized at the C-propeptide domain, followed by triple helix formation in the C to N direction^2^. During this process, pro-α-chains receive significant modifications including hydroxylations of Pro and lysine (Lys) residues forming 3-hydroxyproline (3-Hyp) (at Xaa positions), 4-Hyp, and hydroxylysine (Hyl) residues (both at Yaa positions). Some Hyl residues are further modified by glycosylation, resulting in the formation of galactosyl-hydroxylysine (GHL) and glucosyl-galactosyl-hydroxylysine (GGHL) residues. It should be noted that these modifications occur before triple helix formation, because the responsible enzymes recognize only single stranded substrates.

At least in mammals, procollagen biosynthesis requires further assistance by a unique molecular chaperone, heat shock protein 47 (HSP47), residing in the ER lumen. HSP47 is a member of the serine protease inhibitor (serpin) superfamily and found only in vertebrates. HSP47 specifically binds to the triple helical domain of procollagens^3,4^. The essential role of HSP47 in procollagen biosynthesis was revealed by an HSP47 gene knockout study in which deletion of HSP47 functions caused severe defects in collagen biosynthesis, leading to the embryonic lethal phenotype in mice^5^. Recently, it was also reported that recessive mutations in *hsp47* cause osteogenesis imperfecta (OI) in dachshunds^6^ and humans^7^.

Unlike modification enzymes, HSP47 interacts with triple helical portions of procollagens by recognizing the Gly-Xaa-arginine (Arg) motif. Possible HSP47-binding sites have been estimated to be approximately 30 per procollagen molecule^8^. Triple helical procollagen molecules are transported with HSP47 from the ER to the Golgi apparatus *via* the large coat protein complex II (COPII) of which TANGO1 is involved in the formation^2,9,10^. HSP47 was suggested to be involved in the loading of procollagen into the large COPII vesicles by interacting with TANGO1^11^. However, there are reports on another transport route of procollagen^12^. Furthermore, HSP47 is also reported to bind to decorin, fibromodulin and lumican, which constitute the extracellular matrix, and to be involved in their secretion^13^. HSP47 dissociates from procollagens in a pH-dependent manner at the cis-Golgi or ER-Golgi intermediate compartment (ERGIC), which is transported back to the ER by the ER-retrieval signal at its C-terminus^14–17^.

HSP47 has unique properties among ER-resident molecular chaperones in terms of both transcriptional control and client recognition. In contrast to binding immunoglobulin protein (Bip), glucose regulated protein 94 (Grp94), protein disulfide isomerase (PDI), calnexin, and calreticulin under expression control by the unfolded protein response, HSP47 shows no apparent inducibility by ER stress, but its expression is upregulated by heat stress.

To date, two major roles, both of which are unique for procollagen biosynthesis, have been proposed for HSP47. One is inhibition of lateral aggregation of procollagen molecules in the ER lumen^18^. The other is facilitating procollagen folding by stabilization of triple helical folding intermediates^3,4^. The former is supported by the observation of the aggregated procollagen structure in the ER of embryonic fibroblasts established from *hsp47*−/− mice (*hsp47*−/− MEFs)^18^ and inhibition of collagen-fibril formation in the presence of HSP47 *in vitro*^19^. The latter is also supported by the abnormal properties of collagens secreted from *hsp47*−/− MEFs^5,18^. Furthermore, this function is supported by a report indicating that the mammalian collagen triple helical structure is unstable at body temperature^20^.

To prove the second hypothesis, we determined whether culturing *hsp47*−/− MEF clones at a low temperature compensates for the lack of HSP47 functions and found that all defects in collagens were corrected by lowering the culture temperature to 33 °C. Based on the results, we further discuss the possible role of the chaperone during evolution from poikilothermic invertebrates to homoeothermic vertebrates.

## Results

### Western blot analysis of proteins expressed by *hsp47*+/+ and −/− MEF clones

**Figure 1:**
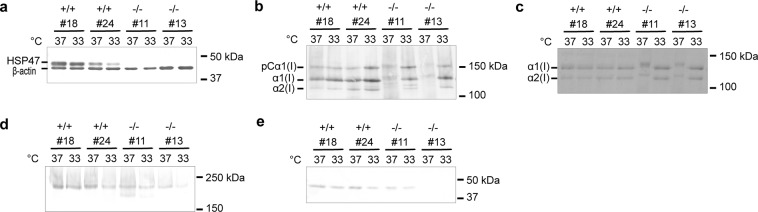
Expression levels of HSP47 in MEF clones and the properties of proteins secreted from the MEF clones. (**a**) Expression levels of HSP47. Proteins extracted from the cell layer were analyzed by western blotting using antibodies against HSP47 and β-actin. Equal amounts of proteins (10 μg) were loaded in each well of the SDS-PAGE gel. (**b**) Collagen secreted from MEF clones. The amount of culture media of MEF clones was divided by the amount of proteins extracted from the cell layer, and the amount of culture media per unit protein amount of the cell layer was calculated. SDS samples were loaded onto SDS-PAGE gels so that the amount of culture media was proportional to this value. The samples were analyzed by western blotting using a collagen-binding peptide^21^. (**c**) Electrophoretic mobility for triple helical region of type I collagen. Culture media of MEFs were digested with 100 μg/mL pepsin at 4 °C for 16 h. Collagens were isolated by salt precipitation and analyzed by SDS-PAGE (5%) under nonreducing conditions. Bands were visualized with Coomassie Brilliant Blue R-250. SDS samples were loaded onto an SDS-PAGE gel as described in (**b**) except for *hsp47*−/− MEF clones. For samples of *hsp47*−/− MEFs, a three-fold higher amount was loaded. (**d**,**e**) Other proteins secreted from MEF clones. Culture media of MEF clones were analyzed by western blotting as described in (**b**). Antibodies against laminin (**d**) and PEDF (**e**) were used.

In this study, we used two lines each of fibroblasts established from wild-type (*hsp47*+/+) and HSP47 gene knockout (*hsp47*−/−) mouse embryos. All MEF clones were able to be maintained at 33 °C as well as 37 °C. The amount of HSP47 expressed in *hsp47*+/+ and −/− MEF clones cultured at 37 °C and 33 °C was compared by western blotting (Fig. 1a). The HSP47 expression in *hsp47*+/+ #18 and #24 was confirmed, and the expression level of HSP47 was slightly decreased at 33 °C in both clones. It was also confirmed that HSP47 was not expressed in *hsp47*−/− #11 or #13. We analyzed the (pro)collagens secreted from the cell lines by western blotting using a collagen-detecting peptide that we developed recently^21^. The electrophoretic mobility of the pCα1(I)-chain and α1(I)-chain was decreased in *hsp47*−/− MEF clones compared with those secreted by *hsp47*+/+ MEF clones at 37 °C (Fig. 1b). This result was essentially consistent with a previous report^22^. The band shifts were normalized by culture at 33 °C and became almost the same as those of *hsp47*+/+ MEF clones. To investigate whether the band shifts found in *hsp47*−/− MEF clones were caused by over-modifications in the triple helical region of collagens, samples were digested with pepsin at 4 °C prior to separation by sodium dodecyl sulfate polyacrylamide gel electrophoresis (SDS-PAGE). As shown in Fig. 1c, the electrophoretic mobility of both α1(I)- and α2(I)-chains was decreased in *hsp47*−/− MEF clones compared with *hsp47*+/+ MEF clones at 37 °C, and the abnormality was also corrected by culture at 33 °C. This result strongly suggests that secretion of over-modified collagens by *hsp47*−/− MEF clones could be normalized by lowering the culture temperature to 33 °C. The phenomenon was found to be specific to collagens, because western blotting results for laminin, another abundant protein in the extracellular matrix, were almost constant regardless of the cell lines and culture temperature (Fig. 1d). We further analyzed the amounts of pigment epithelium-derived factor (PEDF) secreted from the MEF clones. PEDF is a secreted protein and another serpin that binds to the triple helical domain of collagens by recognizing the KGxRGFxGL sequence^23^. Although there is no evidence that PEDF binds to procollagen intracellularly, the interaction between PEDF and procollagen may occur in the ER. Therefore, it may influence the intracellular process of folding and secretion of procollagen like HSP47 does. As shown in Fig. 1e, protein bands corresponding to PEDF were not detected in *hsp47*−/− #13 but were detected in the other tested clones. The amount of collagen-binding protein PEDF was also almost constant regardless of culture temperature and the cell lines except for *hsp47*−/− #13. This result suggests that PEDF did not affect the temperature-dependent alteration of collagen modification found in *hsp47*−/− MEF clones.

### Abnormal collagen localization in *hsp47*−/− MEF clones is corrected by lowering the culture temperature

**Figure 2:**
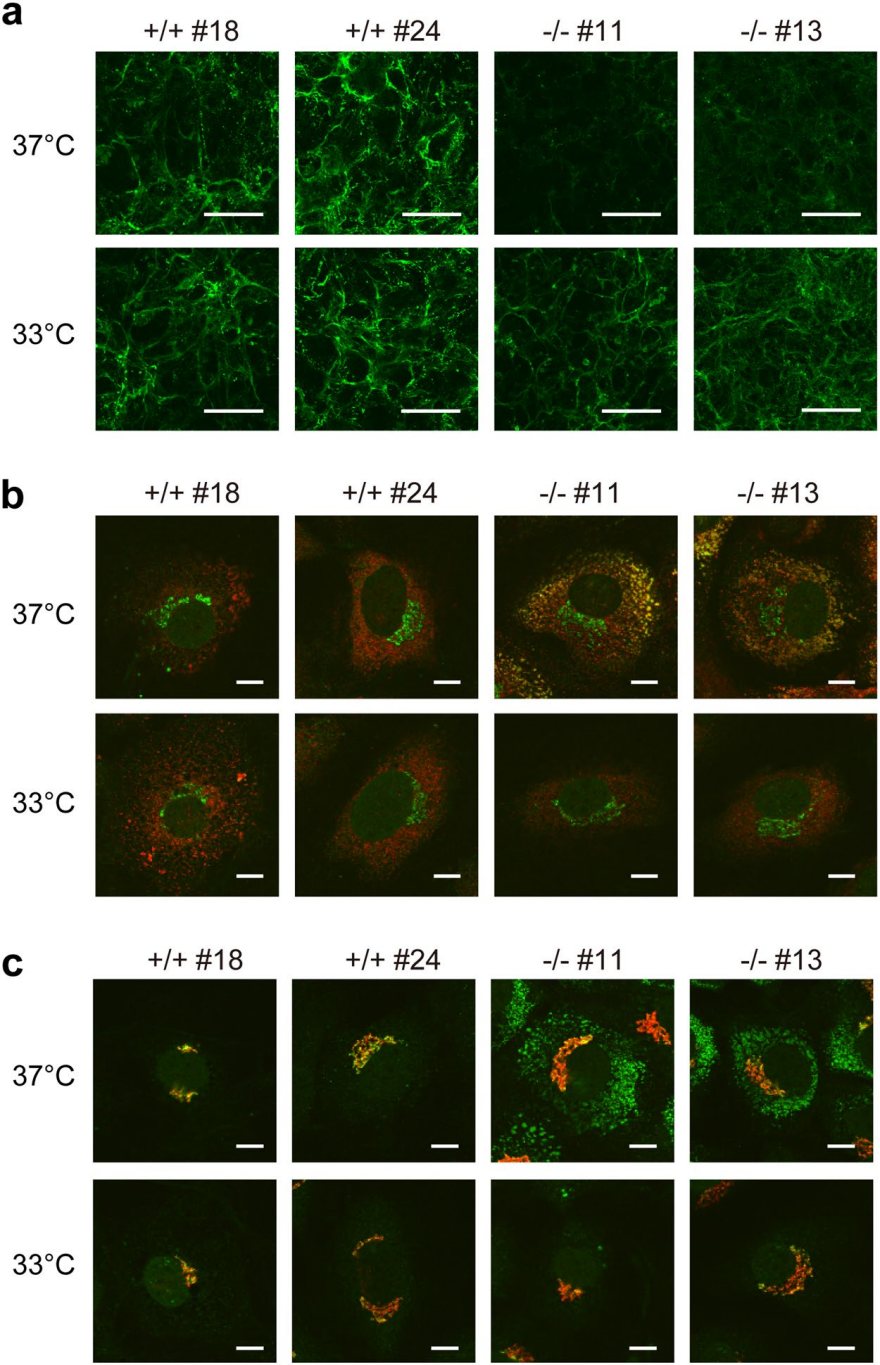
Immunofluorescence staining of extracellular and intracellular type I collagen. (**a**) Immunofluorescence staining of type I collagen secreted from MEF clones was performed with an anti-type I collagen antibody without cell permeabilization. Scale bars: 100 μm. (**b**,**c**) Immunofluorescence staining of permeabilized MEF clones was performed with anti-type I collagen (green) and anti-KDEL antibodies (red) (**b**) or anti-type I collagen (green) and anti-GM130 antibodies (red). (**c**) Scale bars: 10 μm.

The collagen fibril structure around the cultured MEF clones was visualized by immunofluorescence staining using an anti-type I collagen antibody. As shown in Fig. 2a, collagen fibril depositions around *hsp47*−/− MEF clones were thinner and less than those around *hsp47*+/+ MEF clones at 37 °C, which was consistent with a previous report^18^. This abnormality was resolved by culturing the *hsp47*−/− MEF clones at 33 °C. In the case of *hsp47*+/+ MEF clones, no obvious difference in collagen fibril deposition was found at 37 °C and 33 °C. Intracellular localization of procollagens was observed by immunofluorescence staining of permeabilized MEF clones. Double staining was performed with antibodies against type I collagen, ER-retrieval signal sequence (Lys-Asp-Glu-Leu, KDEL) (an ER marker), and GM130 (a Golgi marker). Procollagens accumulated in the ER of *hsp47*−/− MEF clones at 37 °C as reported previously (Fig. 2b)^18^. However, the abnormal accumulation was dissolved at 33 °C. Furthermore, procollagen localization in the Golgi was observed in all cell lines (Fig. 2c). These results indicated that abnormal extracellular collagen fibril formation and procollagen accumulation in the ER of *hsp47*−/− MEF clones were also corrected by lowering the culture temperature to 33 °C.

### α-Chain ratios of collagens secreted from MEF clones

We next investigated whether α-chain ratios of collagens secreted from MEFs were different in *hsp47*+/+ and −/− MEF clones. Culture media of MEF clones were mixed with an internal standard, named stable isotope-labeled collagen (SI-collagen)^24^, and the samples were subjected to salt precipitation and subsequent trypsin digestion. Tryptic marker peptides for quantification of α1(I)-, α2(I)- and α1(III)-chains, and corresponding stable isotopically heavy peptides derived from SI-collagen were analyzed by liquid chromatography mass spectrometry (LC-MS) as reported previously^24^. We calculated the “absolute” amount of each α-chain based on the isotopically light to heavy peak area ratio of the marker peptides by predetermination of the α-chain composition in SI-collagen using known amounts of synthetic nonlabeled marker peptides. In addition, the conformational status of secreted collagens was determined by comparing the α-chain amounts before and after pepsin digestion at 4 °C for 16 h.

**Figure 3:**
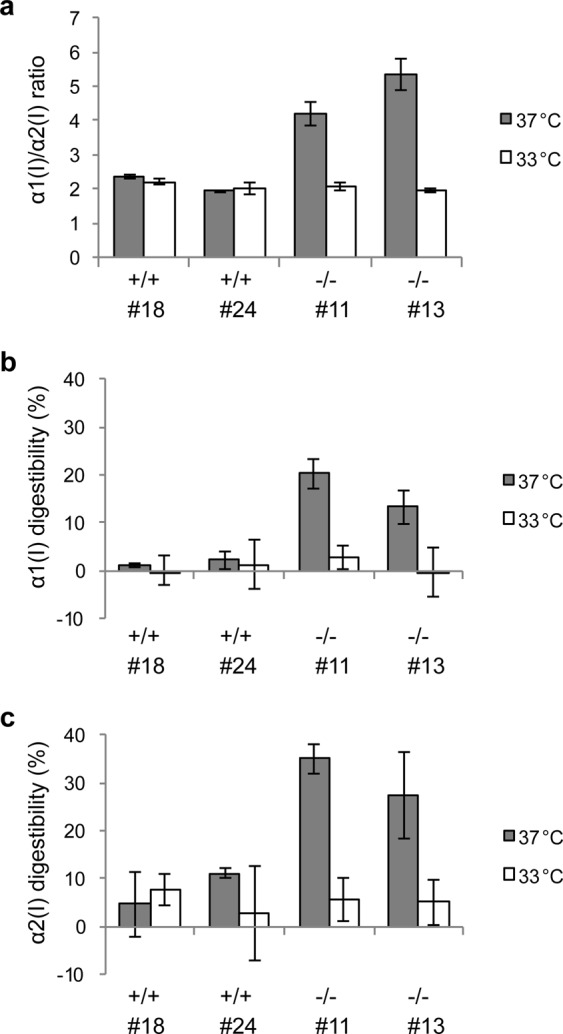
Quantification of type I collagen α-chains secreted from MEF clones. (**a**) Collagen secreted from MEF clones was isolated from culture media by salt precipitation after adding stable isotope-labeled collagen (SI-collagen) as an internal standard. The collagen sample was digested with trypsin after heat denaturation, and generated tryptic marker peptides for quantification of α1(I)- and α2(I)-chains were measured by LC-MS. The α1(I)/α2(I) ratio was calculated based on the result of α-chain quantification. Values are means ± SD (n = 3). (**b**,**c**) The same culture media mixed with SI-collagen in (**a**) were digested with 100 μg/mL pepsin at 4 °C for 16 h. Pepsin-indigestible fractions of α1(I)- and α2(I)-chains were quantified by LC-MS as described in (**a**). Digestibility of α1(I)-chains (**b**) and α2(I)-chains (**c**) were calculated based on the quantitative values for α-chains purified from the pepsinized culture media relative to those for α-chains purified from untreated media analyzed in (**a**). Values are means ± SD (n = 3).

The quantitative values of α-chains are summarized in Supplementary Table S1. The α1(I)-chain to α2(I)-chain ratio was calculated based on the results of α-chain quantification (Fig. 3a). The α1(I)/α2(I) ratios in *hsp47*+/+ MEF clones cultured at both 37 °C and 33 °C were approximately 2. This result indicated that most type I collagen molecules secreted from *hsp47*+/+ MEF clones were [α1(I)]_2_α2(I) heterotrimers. However, the α1(I)/α2(I) ratio in *hsp47*−/− MEF clones cultured at 37 °C was abnormally increased to about 4 or 5, suggesting the existence of homotrimers consisting of three α1-chains. Although the type I collagen molecule is generally a heterotrimer consisting of two α1-chains and one α2-chain, the [α1(I)]_3_ homotrimer is also produced in fetal tissues^25^, fibrous tissues^26–28^, and cancer tissues^29–34^. The ratios of the homotrimers were 42.0±3.9% and 52.5±3.4% (mean ± SD) for clones #11 and #13, respectively. The unusual secretion of [α1(I)]_3_ homotrimers by *hsp47*−/− MEF clones was stopped by culture at 33 °C, and the α1(I)/α2(I) ratio was recovered to 2.

The conformational status of secreted type I collagen at 4 °C was evaluated by examining susceptibility to pepsin digestion. Ratios of pepsin-digestible collagen α-chains were calculated based on the quantitative values of the α-chains purified from untreated culture media and pepsin-treated media (Fig. 3b,c). When MEF clones were cultured at 37 °C, the ratios of pepsin-digestible α-chains were higher in *hsp47*−/− MEF clones than *hsp47*+/+ MEF clones. This result indicated that collagens with a loose or misaligned triple helix were secreted from *hsp47*−/− MEF clones at 37 °C, which is consistent with a previous study^5^. The abnormally high ratios of digestible α-chains were reduced to less than 10% at 33 °C. However, the pepsin digestibility of each α-chain in *hsp47*+/+ MEF clones did not differ between culture at 37 °C and 33 °C, and only small proportions (~10%) of both α-chains were digested by pepsin. In addition, the α2(I)-chain showed higher digestibility than the α1(I)-chain at 37 °C in all cell lines but especially in *hsp47*−/− MEF clones. This result indicated that the ratio of secreted unfolded and/or misfolded collagens sensitive to pepsin digestion was higher for [α1(I)]_2_α2(I) heterotrimers than [α1(I)]_3_ homotrimers.

### Post-translational modifications of type I collagen

The results in Fig. 1b and c suggest that excessive post-translational modifications occurred in the triple helical domain of type I collagen secreted from *hsp47*−/− MEF clones at 37 °C. The modifications were suggested to include prolyl 3- and 4-hydroxylations, and lysyl hydroxylation, which were often accompanied by further glycosylation. Therefore, we analyzed the difference in post-translational modifications among cell lines and shifts in modifications according to the culture temperature.

**Figure 4:**
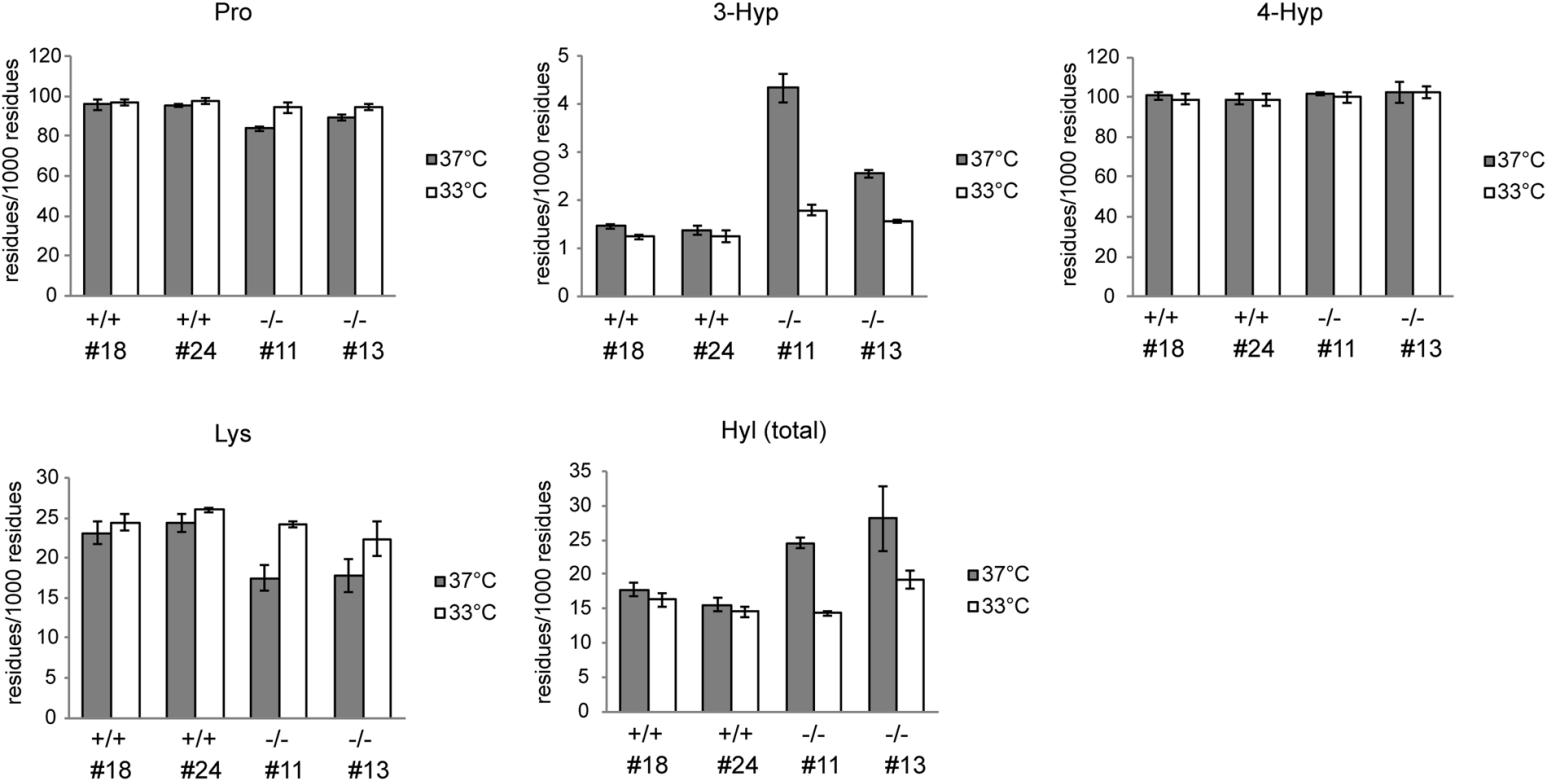
Quantification of total post-translational modifications in type I collagen. After culture media of MEF clones were digested with 100 μg/mL pepsin at 4 °C for 16 h, collagens were isolated by salt precipitation. SI-collagen was mixed into the purified collagen as an internal standard, and the sample was subjected to SDS-PAGE. Proteins were transferred onto a PVDF membrane, and the membrane was stained with Coomassie Brilliant Blue R-250. The region containing both α1(I)- and α2(I)-chains was excised from the membrane for acid hydrolysis. Pro, Lys, and their post-translational modifications were quantified by LC-MS. The contents of respective amino acids are expressed as residues/1000 residues. Values are means ± SD (n = 3).

We performed direct acid hydrolysis of type I collagen electroblotted onto a polyvinylidene fluoride (PVDF) membrane after separation by SDS-PAGE^35^. We mixed SI-collagen into the sample before SDS-PAGE, and the region containing both α1(I) and α2(I) was excised from the electroblotted membrane for gas phase acid hydrolysis. Pro, 3-Hyp, 4-Hyp, Lys, and total Hyl (Hyl+GHL+GGHL) were quantified by LC-MS with correction using corresponding stable isotopically heavy analytes as internal standards to calculate the number of residues per 1000 residues of the triple helical region (Fig. 4 and Supplementary Table S2).

In collagens secreted from both *hsp47*−/− MEF clones at 37 °C, 3-Hyp and Hyl residues were increased compared with those from *hsp47*+/+ MEF clones, and their substrates, unmodified Pro and Lys residues, were decreased accordingly. These over-modifications were suppressed at 33 °C to the same level as that in *hsp47*+/+ MEF clones. In addition, the content of 4-Hyp residues was approximately 100 residues/1000 residues and almost constant in all samples, indicating that most Pro residues at Yaa positions had been converted to 4-Hyp residues. As a result of calculating the ratio of 4-Hyp residues to the total Pro residues, 4-Hyp rate was slightly higher in collagens secreted from *hsp47*−/− MEF clones at 37 °C. The abnormal 4-Hyp rate was suppressed at 33 °C (Supplementary Table S3).

We further examined the effects of HSP47 deficiency and culture temperature on prolyl 3-hydroxylation and lysyl hydroxylation/glycosylation at specific sites in type I collagen by LC-MS analysis of tryptic peptides containing the modification sites. Although prolyl 3-hydroxylation at α1(I)/α2(I) Pro716 and Pro719 were recently identified to be specific for tendons^36^, we detected 3-Hyp at the sites in skin fibroblast-derived collagens. This result was probably due to the difference in prolyl 3-hydroxylation between collagens produced in cell culture and those in tissues as suggested previously^37^. A marked increase of prolyl 3-hydroxylation was found in the consecutive modifications sites, α1(I)/α2(I) Pro707, Pro716, and Pro719, of *hsp47*−/− MEF clones cultured at 37 °C (Supplementary Fig. S1a). These over-modifications were shifted at 33 °C to a similar level as that in *hsp47*+/+ MEF clones. However, 3-hydroxylation at α1(I) Pro986, the absence of which is associated with OI^38^, was almost completed in all samples.

Post-translational Lys modifications were also quantified at eight known sites (Supplementary Fig. S1b). The Lys hydroxylation and following glycosylation to form GHL and GGHL residues were increased at the specific modification sites except for almost fully hydroxylated (α2(I) Lys87) or glycosylated (α1(I) Lys87) sites in both *hsp47*−/− MEF clones cultured at 37 °C compared with those in *hsp47*+/+ MEF clones. The excessive modifications were also shifted to the normal level at 33 °C.

### Measurement of the melting temperature of [α1(I)]_2_α2(I) heterotrimers

**Figure 5:**
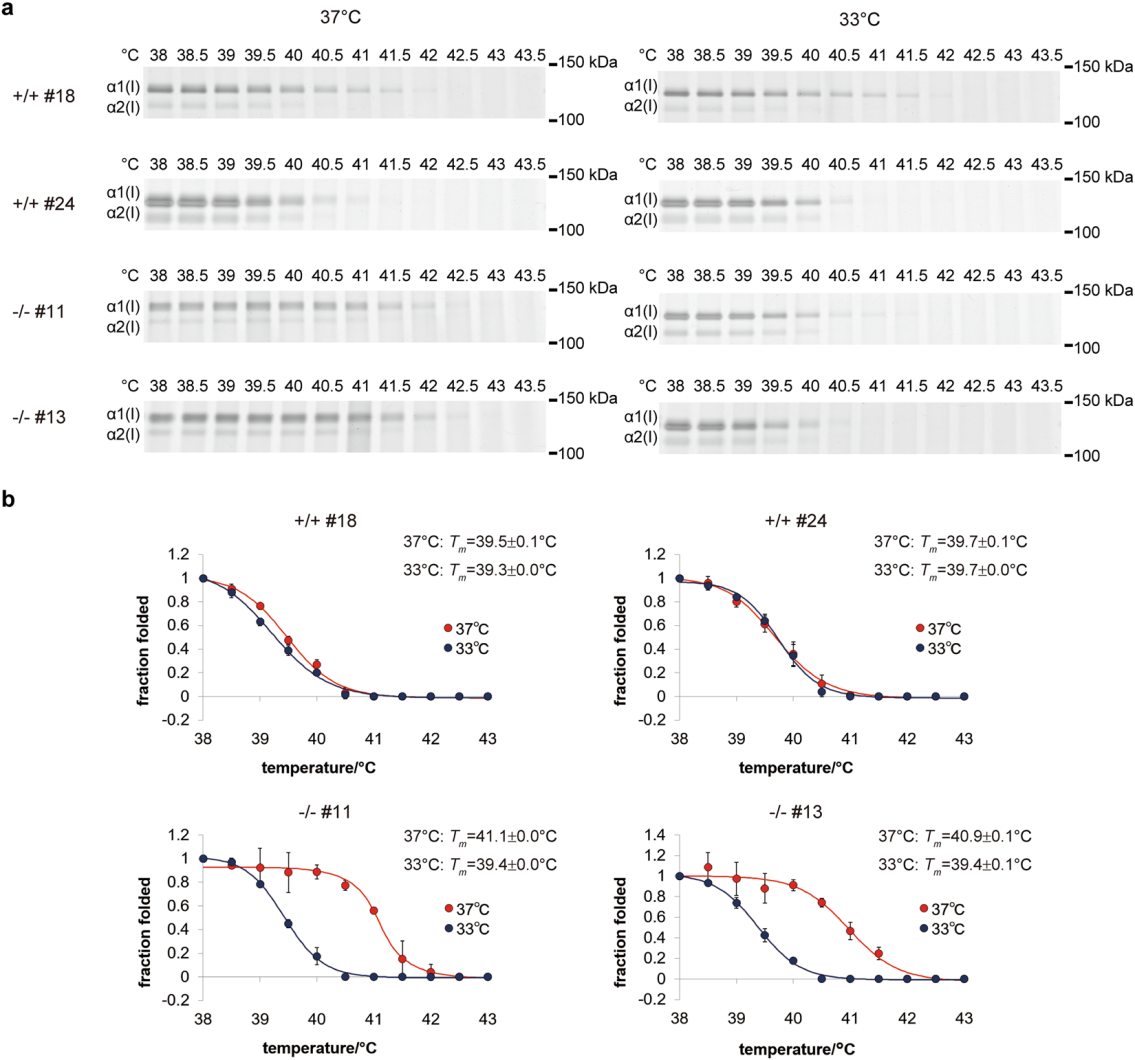
Measurement of the melting temperature of [α1(I)]_2_α2(I) heterotrimers. (**a**) Melting temperature of collagens secreted from MEF clones was determined by susceptibility to trypsin/chymotrypsin digestion. After the culture media of MEF clones were digested with 100 μg/mL pepsin at 4 °C for 16 h, collagens were isolated by salt precipitation. The purified collagen solution was heated and then digested with trypsin (100 μg/mL) and chymotrypsin (250 μg/mL) for 2 min at room temperature. SDS-PAGE (5% gel) was performed under nonreducing conditions, and the bands were visualized by silver staining. (**b**) The band intensity of the α2(I)-chain at each temperature in (**a**) and additional two technical replicates were measured with image analysis software ImageJ, and four parameter logistic curve fitting was carried out. Graphs were plotted so that the fraction folded at 38 °C was 1. The temperature at which the fraction folded was 0.5 is shown as the melting temperature of [α1(I)]_2_α2(I) heterotrimers. Values are means ± SD (n = 3).

Post-translational modifications in triple helix-forming regions likely alter the thermal stability of collagen, as prolyl 4-hydroxylation is known to stabilize the triple helical structure^1^. Prolyl 3-hydroxylation may increase the thermal stability of the collagen triple-helix^39^, but the effect of lysyl hydroxylation and further glycosylation are controversial^40–42^. Because the *hsp47*−/− MEF clones secreted over-modified collagens at 37 °C (Fig. 4, Supplementary Fig. S1 and Supplementary Table S3), we estimated the thermal stability of the type I collagen (α1)_2_α2 heterotrimer produced by each MEF clone at either 37 °C or 33 °C. Triple helical collagen samples were prepared from conditioned media by salt precipitation after pepsin treatment at 4 °C for 16 h. The purified collagen samples were heated at different temperatures, followed by trypsin/chymotrypsin digestion at 20 °C. After separation by SDS-PAGE, melting temperatures (*T*_*m*_) for [α1(I)]_2_α2(I) heterotrimers were determined by quantifying the band intensities corresponding to the α2(I)-chain with image analysis software (Fig. 5). It should be mentioned that *T*_*m*_ values estimated by this method are apparent values that do not indicate absolute values for the denaturation temperatures. The *T*_*m*_ values for [α1(I)]_2_α2(I) heterotrimers secreted from *hsp47*+/+ MEF clones were not significantly different at both culture temperatures. This result was consistent with the steady post-translation levels of the collagens (Fig. 4, Supplementary Fig. S1 and Supplementary Table S3). In contrast, *T*_*m*_ values for collagens secreted from *hsp47*−/− MEF clones at 37 °C were higher than those for collagens secreted from *hsp47*+/+ MEF clones. The enhancement of thermal stability at 37 °C should reflect the over-modifications of the corresponding collagen samples (Fig. 4, Supplementary Fig. S1 and Supplementary Table S3). This higher thermal stability was resolved by culturing the *hsp47*−/− MEF clones at 33 °C. In addition, the bands for the α1(I)-chain remaining even at a higher temperature than those for α2(I) in Fig. 5a were considered to be due to the higher thermal stability of the [α1(I)]_3_ homo-triple helix^43,44^.

## Discussion

In the absence of HSP47, various defects in collagen biosynthesis were observed at the normal culture temperature of 37 °C. The ratio of secreted unfolded and/or misfolded collagens sensitive to protease digestion was increased. Secreted triple helical molecules showed higher *T*_*m*_ values compared with wild-type cells, which was caused by over-modifications. Abnormal accumulation of procollagens and their folding intermediates in the ER as well as less and thinner fibril depositions around the cells were also observed. These abnormalities caused by the lack of HSP47 were essentially consistent with those reported previously^5,7,18,22^. In addition, *hsp47*−/− MEF clones secreted [α1(I)]_3_ homotrimers at a higher ratio. This is probably because of the contribution of [α1(I)]_3_ homotrimers with higher thermal stability of the triple helix than [α1(I)]_2_α2(I) heterotrimers^43,44^. All abnormalities in collagens were shown to be corrected by lowering the culture temperature by 4 °C. This finding indicates that HSP47 is responsible for resolving the heat-related problems in procollagen biosynthesis, which is supported by the heat shock-inducibility of HSP47 expression.

The biosynthetic process of procollagens is unique in terms of protein folding. Procollagens have neither a hydrophobic core nor intra-main chain hydrogen bonds that lead molecular compacting during their folding. Furthermore, the thermal stability of the triple helix is acquired by significant post-translational modifications. Thus, the thermal stability is not solely ensured by the amino acid sequences encoded by the genes. Prolyl 4-hydroxylation, which occurs on most Pro residues in Gly-Xaa-Pro triplets, most greatly contributes to stabilization of the triple helical structure^1^. In addition, other modifications, such as prolyl 3-hydroxylation and modifications on lysine side-chains, may affect the thermal stability^39,40,42^. Because modification enzymes recognize only single chain sites as their substrates, the modifications should continue until triple helix formation completes. The existence of such an autonomous feedback system to ensure thermal stability of the triple helix is supported by a report in which over-modified collagen was secreted from human skin fibroblasts cultured at a high temperature of 40.5 °C^45^. The over-modifications found in *hsp47*−/− MEF clones also reflected a delay or defect in procollagen folding in the ER. However, the increase of *T*_*m*_ values for *hsp47*−/− MEF clones was as small as about 1.5 °C, and secreted collagens were sensitive to protease digestion. Most procollagen molecules secreted from *hsp47*−/− MEF clones failed to acquire enough thermal stability at 37 °C (Figs. 3b,c and 5), even if they received over-modifications. This observation is consistent with a report showing that the mammalian type I collagen molecule itself does not have enough thermal stability at body temperature^20^. Our results strongly support the hypothesis that HSP47 achieves procollagen folding by stabilizing its triple helical conformation, which is otherwise unstable at body temperature.

These data suggest that 33 °C is a low enough temperature to realize procollagen during procollagen biosynthesis, but other possible factors that contribute to the stabilization of the triple helix are considered. PEDF and FK506-binding protein FKBP65 also bind to correctly folded procollagen^46^. PEDF is another serpin that binds to the collagen triple helix^23^. It is a secreted protein and there is no evidence that PEDF binds to procollagen intracellularly, but PEDF may interact with procollagen in the ER. *Hsp47*-null mice show an embryonic lethal phenotype. These proteins cannot compensate for the function of HSP47 at 37 °C. In addition, the secretion amount of PEDF was almost constant regardless of culture temperature (Fig. 1e), and the role of PEDF is unlikely to change significantly at 33 °C. Although there is the possibility that proteins other than HSP47 stabilize procollagen at 33 °C, low temperature is probably the main factor that stabilizes the procollagen triple helical structure in *hsp47*−/− MEF clones.

No obvious staining of procollagen aggregation was observed even in the ER of *hsp47*−/− MEF clones cultured at 33 °C (Fig. 2b,c). This result suggests that inhibition of the aggregation caused by lateral association of folded procollagen molecules in the ER is not essential in the biosynthetic pathway. In addition, the shape of extracellular collagen fibrils around *hsp47*+/+ MEF clones showed little difference at 37 °C and 33 °C (Fig. 2a), so there may be no significant difference in the ability of lateral association of procollagen in the ER between the temperatures.

TANGO1 is a protein involved in the formation of large COPII vesicles that can carry procollagen^9,10^. *Tango1*-null mice are known to have abnormal bone formation due to delayed secretion of collagen^47^. Large COPII vesicles are formed at ER exit sites (ERES) and packs TANGO1 with procollagen^10^. In addition to vesicular transport, TANGO1 is involved in the formation of tunnels from ERES to Golgi for procollagen transport^12^. TANGO1 was also reported to bind to HSP47^11^. This binding is thought to contribute to concentrating procollagen around TANGO1. In this study, *hsp47*−/− MEF clones secreted type I collagen at the same level as *hsp47*+/+ MEF clones at 33 °C (Fig. 2a and Supplementary Table S1a). Thus, it is unlikely that HSP47 is important for collagen secretion as long as the triple helix of procollagen forms normally. Recently, ERES-microautophagy model was suggested^48^. After the misfolded procollagen enters ERES, the ERES decorated with LC3 and COPII is engulfed by lysosomes and degraded. The interaction between HSP47 and TANGO1 might contribute to selecting procollagen for autophagy or secretion. HSP47 was also reported to function as a regulator of the ER stress response signaling protein inositol-requiring enzyme 1α (IRE1α)^49^. Further investigation is needed on additional possible functions of HSP47.

All animals have collagens, whereas HSP47 exists only in vertebrates. The chaperone function of HSP47 is essential in mammals, although invertebrates are able to synthesize collagen without the assistance of HSP47. Therefore, mammalian collagens have evolved without acquiring their own thermal stability. HSP47 might have facilitated molecular evolution of collagens by enhancing apparent thermal stability of the triple helix. Molecular diversity and/or mutations in collagens accompanied by loss of thermal stability might have been tolerated by the action of HSP47. In fact, in *hsp47*−/− MEF clones, defects in the type I collagen (α1)_2_α2 heterotrimer, a more evolved molecule, were more severe, and an increase in the ratio of the (α1)_3_ homotrimer, a prototype molecule, was also found (Fig. 3A). In addition, fish type I collagen has three different α-chains^50^. Possibly, HSP47 has facilitated collagen evolution by acquiring molecular diversity. This hypothesis is essentially the same as that proposed for HSP90 by Lutherford and Lindquist^51^. HSP90 has been suggested to act as a capacitor for molecular evolution by masking mutations accompanied by loss of conformational stability in client proteins. HSP47 and HSP90 are particular chaperones that bind to and stabilize correctly folded conformations and hence might have contributed to molecular evolution of client proteins. It is also possible that HSP47 has enabled molecular adaptation to a higher body temperature, which led the evolution from poikilotherms to homeotherms.

## Materials and Methods

### Cell culture

*Hsp47*+/+ and −/− MEF clones have been reported previously^5^. The cells were cultured in Dulbecco’s modified Eagle’s medium (Wako Pure Chemical Industries, Osaka, Japan) supplemented with 10% fetal bovine serum (Thermo Fisher Scientific, Waltham, MA), 100 U/mL penicillin, and 100 μg/mL streptomycin (Sigma-Aldrich, St. Louis, MO) at 37 °C in a 5% CO_2_ atmosphere. To perform all experiments, the medium was replaced with human fibroblasts defined medium (HFDM)-1(+) (Cell Science & Technology Institute Inc., Miyagi, Japan) containing 100 U/mL penicillin and 100 μg/mL streptomycin after the cells had reached confluency. The cells were maintained at 37 °C or 33 °C in a 5% CO_2_ atmosphere.

### SDS-PAGE

MEF clones were cultured in HFDM-1(+) for 3 days after the cells had reached confluency in 9-cm dishes. After the culture media were centrifuged (2,290 × *g*, 4 °C, 15 min), the supernatants were digested with pepsin (100 μg/mL) in 0.1 N HCl at 4 °C for 16 h. Collagens were isolated by salt precipitation (1 M NaCl/0.1 N HCl) for 3 h at 4 °C, dissolved in SDS-PAGE sample buffer (50 mM Tris-HCl, pH 6.7, 10% glycerol, 2% SDS, and 0.002% bromophenol blue), and heated at 95 °C for 5 min. SDS-PAGE (5% gels) was performed under nonreducing conditions, and protein bands were visualized with Coomassie Brilliant Blue R-250.

### Western blotting

MEF clones were cultured in HFDM-1(+) for 3 days after the cells had reached confluency in 9-cm dishes. After collecting the culture media, the cells were washed with phosphate-buffered saline (PBS) and treated with lysis buffer [50 mM Tris-HCl, pH 8, 150 mM NaCl, 5 mM EDTA, 0.1% Nonidet P-40, 2 mM N-ethylmaleimide, 1 mM phenylmethylsulfonyl fluoride (PMSF), 1 μg/mL leupeptin, and 1 μg/mL pepstatin A] on ice for 15 min. After centrifugation (20,600 × *g*, 4 °C, 15 min), soluble proteins in the supernatants were quantified by the Bradford method. The supernatants and collected culture media were mixed with 5 × SDS sample buffer and heated at 95 °C for 5 min. Cell lysate samples with equal protein quantities were applied to gels. Medium samples were applied to gels to be proportional to the amount of culture media per unit protein amount of the cell layer. SDS-PAGE was performed using 5% or 10% gels in the presence of 91 mM 1,4-dithiothreitol. After transfer to a nitrocellulose membrane, non-specific binding sites were blocked with 5% dry skim milk/Tris-buffered saline (TBS; 50 mM Tris-HCl, pH 7.4, and 150 mM NaCl) for 1 h. After washing with TBS, the membrane was treated with a primary antibody diluted in 2% dry skim milk/TBS or the collagen-detecting peptide/PBS^21^ and washed again with TBS. After treatment with a secondary antibody diluted in 2% dry skim milk/TBS, the membrane was washed with TBS containing 0.1% Tween-20. To detect HSP47, β-actin, and PEDF, bands developed using a Pierce western blotting substrate kit (Thermo Fisher Scientific) were analyzed with a LAS-3000 CCD imager (Fujifilm, Tokyo, Japan). For collagen and laminin detection, bands were visualized using an alkaline phosphatase conjugate substrate kit (Bio-Rad Laboratories, Hercules, CA).

Antibodies were an anti-laminin rabbit polyclonal antibody (ab11575, Abcam, Cambridge, MA), anti-PEDF mouse monoclonal antibody (KM037, Transgenic, Kumamoto, Japan), anti-HSP47 mouse monoclonal antibody (SPA-470, StressGen Biotechnologies, San Diego, CA), anti-β-actin monoclonal antibody (A5316, Sigma-Aldrich), goat anti-rabbit IgG-AP conjugate (sc-2034, Santa Cruz Biotechnology, Dallas, TX), and goat anti-mouse IgG-HRP conjugate (W402B, Promega, Fitchburg, WI).

### Immunofluorescence staining

After MEF clones had reached confluency in 35-mm glass bottom dishes, the media were replaced with HFDM-1(+). The cells were cultured for 1 day for extracellular staining or 3 days for intracellular staining. After washing with PBS, the cells were fixed with 4% formaldehyde/PBS for 15 min. For intracellular staining, cells were washed with PBS and incubated with 0.5% Triton X-100/PBS for 15 min. After washing with PBS, blocking was performed with 3% bovine serum albumin (BSA)/PBS for 1 h. Then, the cells were incubated with primary antibodies diluted in 1% BSA/PBS, washed with PBS, and incubated with secondary antibodies diluted in 1% BSA/PBS. After washing with PBS, the cells were observed under an FV-1000 confocal laser microscope (Olympus, Tokyo, Japan).

Antibodies used for extracellular staining were an anti-collagen type I rabbit polyclonal antibody (600-401-103-0.1, Rockland Inc., Gilbertsville, PA) and goat anti-rabbit IgG (H+L) antibody, FITC conjugate (65-6111, Thermo Fisher Scientific). Antibodies used for intracellular staining were an anti-mouse collagen type I rabbit polyclonal antibody (AB765P, Merck Millipore, Billerica, MA), anti-KDEL mouse monoclonal antibody (10C3) (ADI-SPA-827, Enzo life Sciences, Farmingdale, NY), anti-GM130 mouse monoclonal antibody (610822, BD), goat anti-rabbit IgG (H+L) antibody, FITC conjugate (65-6111, Thermo Fisher Scientific), and goat anti-mouse IgG (H+L) antibody, Alexa Fluor 594 conjugate (ab150116, Abcam)

### Quantification of collagen α-chains by LC-MS

The MEF clones were cultured in HFDM-1(+) for 3 days after the cells had reached confluency in 9-cm dishes. The cells were washed with PBS and treated with lysis buffer on ice for 15 min. After centrifugation (20,600 × *g*, 4 °C, 15 min), soluble proteins in the supernatants were quantified by the Bradford method. The collected culture medium was acidified with HCl (final concentration: 0.1 N) and mixed with SI-collagen that was prepared in a culture of human embryonic lung fibroblasts^24^. The culture medium was then divided into two aliquots. One aliquot was digested with pepsin (100 μg/mL; Sigma-Aldrich) at 4 °C for 16 h, while the other was stored at 4 °C without pepsin treatment. Collagens were isolated from the culture media by salt precipitation (1 M NaCl) for 1 h on ice. The collagen sample was digested with sequencing grade-modified trypsin (Promega) at 37 °C for 16 h in 100 mM Tris-HCl/1 mM CaCl_2_ (pH 7.6) after heating at 60 °C for 30 min. The tryptic digest was analyzed by LC-triple quadrupole (QqQ)-MS on a hybrid QqQ/linear ion trap 3200 QTRAP mass spectrometer (AB Sciex) coupled to an Agilent 1200 Series HPLC system (Agilent Technologies) using a BIOshell A160 Peptide C18 HPLC column (5 µm particle size, L × I.D. 150 mm × 2.1 mm; Supelco) as described previously^24^. Marker peptides established in a previous study^24^ for α1(I)-chain (GVQGPOGPAGPR and GVVGLOGQR; O indicates 4-Hyp), α2(I)-chain (EGPVGLOGIDGR and GPSGPQGIR), and α1(III)-chain (GROGLOGAAGAR and GLAGPOGMOGPR) were detected in multiple reaction monitoring (MRM) mode. The molar concentrations of α1(I)-, α2(I)- and α1(III)-chains were calculated by the peak area ratio of the marker peptides relative to stable isotopically heavy peptides derived from SI-collagen. The absolute concentrations of respective α-chains in SI-collagen were predetermined by MRM analysis using corresponding nonlabeled synthetic peptides (Supplementary Fig. S2 and Supplementary Table S4) as internal standards of known concentrations.

### Quantification of total levels of post-translational modifications in collagens by LC-MS

MEF clones were cultured in HFDM-1(+) for 3 days after the cells had reached confluency in 9-cm dishes. Collagens were purified from the collected culture media by immobilized pepsin digestion (0.1 mg/mL in 0.1 N HCl; Sigma-Aldrich) and salt precipitation (1 M NaCl)^24^. SI-collagen was mixed into the purified collagens as an internal standard, and then the sample was subjected to SDS-PAGE under nonreducing conditions using a 5% gel. The gel was electroblotted onto a PVDF membrane at 215 mA for 90 min. The membrane was stained with Coomassie Brilliant Blue R-250, and the region containing both α1(I)- and α2(I)-chains was excised from the membrane. After washing twice with 10% acetic acid/40% methanol for 5 min, the excised membrane was placed in a PicoTag sample tube (Waters) and subjected to acid hydrolysis (6 N HCl/1% phenol, 110 °C for 20 h in the gas phase under N_2_). The acid hydrolysate of collagens was extracted from the membrane by 0.1% acetic acid/5 mM ammonium acetate in 50% acetonitrile and analyzed by LC-QqQ-MS using a ZIC-HILIC column (3.5 µm particle size, L × I.D. 150 mm × 2.1 mm; Merck Millipore) as described previously^52^. Pro, Lys, Arg, 3-Hyp, 4-Hyp, and Hyl were detected in MRM mode. The contents of respective amino acids expressed as residues/1000 total residues were calculated by the peak area ratio of the analytes relative to stable isotopically heavy analytes derived from SI-collagen according to the following formula: (light/heavy) / (light Arg/heavy Arg)^24^.

### Determination of apparent *T*_*m*_ values for secreted collagens

MEF clones were cultured in HFDM-1(+) for 3 days. After the collected culture media were centrifuged (2,290 × *g*, 4 °C, 15 min), the supernatants were digested with pepsin (100 μg/mL) in 0.1 N HCl at 4 °C for 16 h. Collagens were isolated by salt precipitation (1 M NaCl/0.1 N HCl) for 3 h at 4 °C and dissolved in 50 mM Tris-HCl (pH 7.4) containing 150 mM NaCl, 10 mM EDTA, and 1% (v/v) Triton X-100. This solution was dispensed into PCR tubes, and the temperature was gradually increased from 38 °C to 43.5 °C by 0.5 °C/5 min in a thermal cycler. After maintaining the target temperature for 5 min, the PCR tube was removed from the thermal cycler and cooled at 20 °C for 1 min. The solution was digested with trypsin (100 μg/mL) and chymotrypsin (250 μg/mL) for 2 min at 20 °C. The reaction was stopped by addition of PMSF at a final concentration of 1 mM, followed by addition of 5 × SDS-PAGE sample buffer. SDS-PAGE (5% gel) was performed under nonreducing conditions, and the bands of proteins were visualized by silver staining using a sil-best stain one kit (Nacalai Tesque, Kyoto, Japan). The band intensity of the α2(I)-chain at each temperature was measured by image analysis software ImageJ^53^.

## Supplementary information

Supplementary information
