# Supplementary material for: Lowering the culture temperature corrects collagen abnormalities caused by HSP47 gene knockout
Source: Sci Rep. 2019 Nov 22;9:17433. doi: 10.1038/s41598-019-53962-0 (PMC6874656; doi:10.1038/s41598-019-53962-0)
Supplement: Supplementary file 1 — Supplementary information [file 41598_2019_53962_MOESM1_ESM.docx]

Supplementary information

**Lowering the culture temperature corrects collagen abnormalities caused by HSP47 gene knockout**

Kazunori K. Fujii^1,2^, Yuki Taga^2,3^, Takayuki Sakai^1^, Shinya Ito^4^, Shunji Hattori^3^, Kazuhiro Nagata^4^ and Takaki Koide^1,*^

^1^Department of Chemistry and Biochemistry, School of Advanced Science and Engineering, Waseda University, Shinjuku, Tokyo 169-8555, Japan; ^2^ These authors contributed equally; ^3^ Nippi Research Institute of Biomatrix, 520-11 Kuwabara, Toride, Ibaraki 302-0017, Japan; ^4^ Laboratory of Molecular and Cellular Biology, Faculty of Life Sciences, Kyoto Sangyo University, Kamigamo, Kita-ku, Kyoto 803-8555, Japan.

Address correspondence to: Takaki Koide, Department of Chemistry and Biochemistry, School of Advanced Science and Engineering, Waseda University, Shinjuku, Tokyo 169-8555, Japan.

Phone/Fax: +81-3-5286-2569; E-mail: [koi@waseda.jp](mailto:koi@waseda.jp)

**Materials and Methods**

**Site-specific analysis of Pro and Lys modifications in type I collagen by LC-MS**

The relative abundance of Pro and Lys modifications at specific modification sites was semiquantitatively estimated by LC-MS as described previously (1). In brief, the purified collagen sample was digested with trypsin following heat denaturation as described for quantification of collagen α-chains. The tryptic digest was then subjected to LC-quadrupole time-of-flight (QTOF)-MS on an ultra-high resolution QTOF mass spectrometer (maXis II; Bruker Daltonics) coupled to a Shimadzu Prominence UFLC-XR system (Shimadzu) using an Ascentis Express C18 HPLC column (5 µm particle size, L × I.D. 150 mm × 2.1 mm; Supelco). The relative abundance of Pro 3-hydroxylation (Pro or 3-hydroxyproline (3-Hyp)) and Lys modifications (Lys, hydroxylysine (Hyl), galactosyl-hydroxylysine (GHL), or glucosyl-galactosyl-hydroxylysine (GGHL)) at each modification site was calculated by the peak area ratios of peptides containing the respective molecular species.

**Profiles of the synthesized peptides**

Peptide chains were constructed by the 9-fluorenylmethoxycarbonyl-based solid phase method on Wang resin. The protected peptide resins were treated with trifluoroacetic acid/H_2_O/*m*-cresol/thioanisole/1,2-ethanedithiol (82.5/5/5/5/2.5, v/v) for 2 or 4 h at room temperature. The peptides were purified by Shimadzu reverse phase-high performance liquid chromatography (RP-HPLC) and identified by Autoflex III MALDI-TOF MS (Bruker Daltonics).


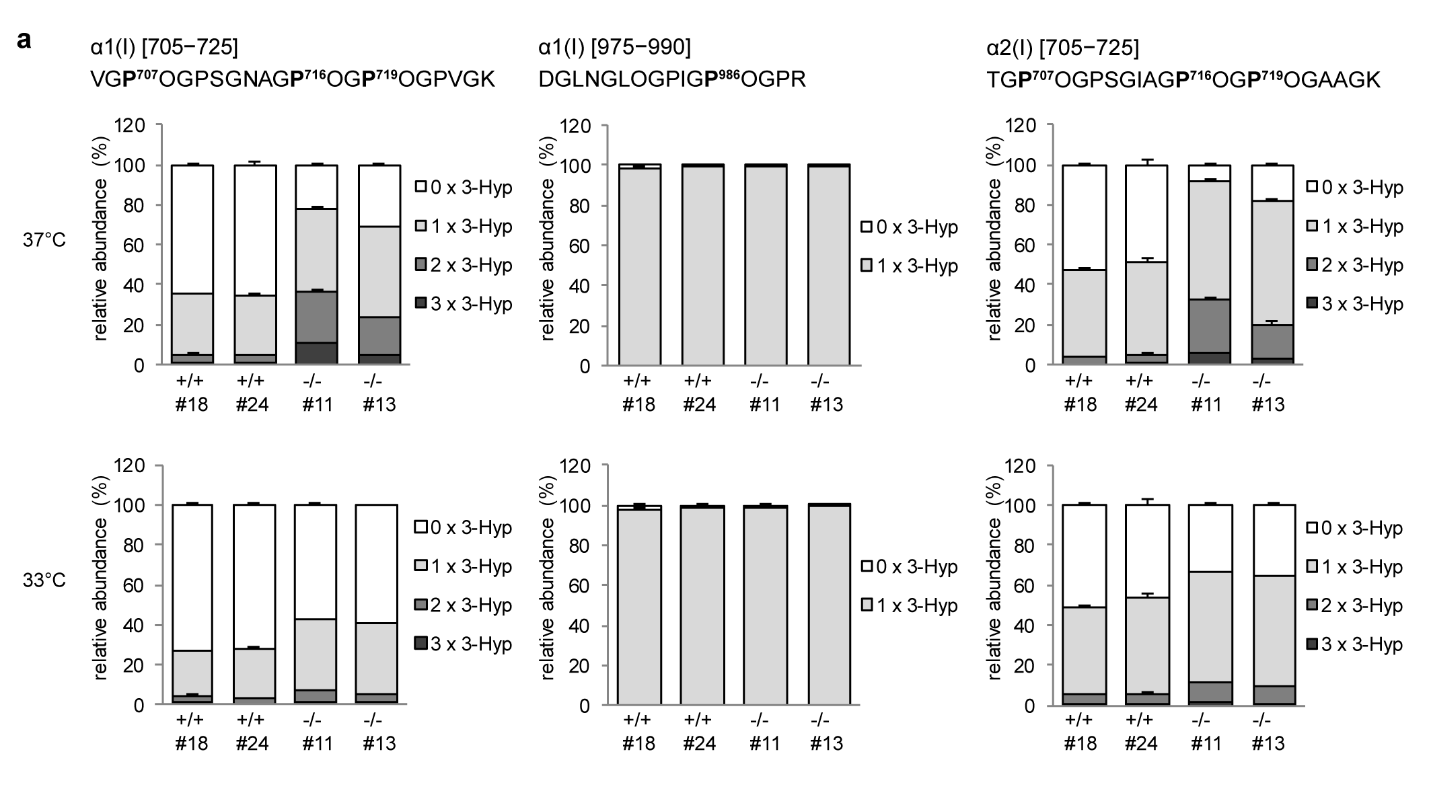


**Figure S1.** **Quantification of site-specific post-translational modifications in type I collagen.**

(a, b) Secreted collagens were digested with trypsin at 37°C for 16 h following heat denaturation at 60°C for 30 min. Prolyl 3-hydroxylation (a) and Lys post-translational modifications (b) at specific sites were quantified by LC-MS. Values are means ± SD (n = 3). O indicates 4-hydroxyproline.


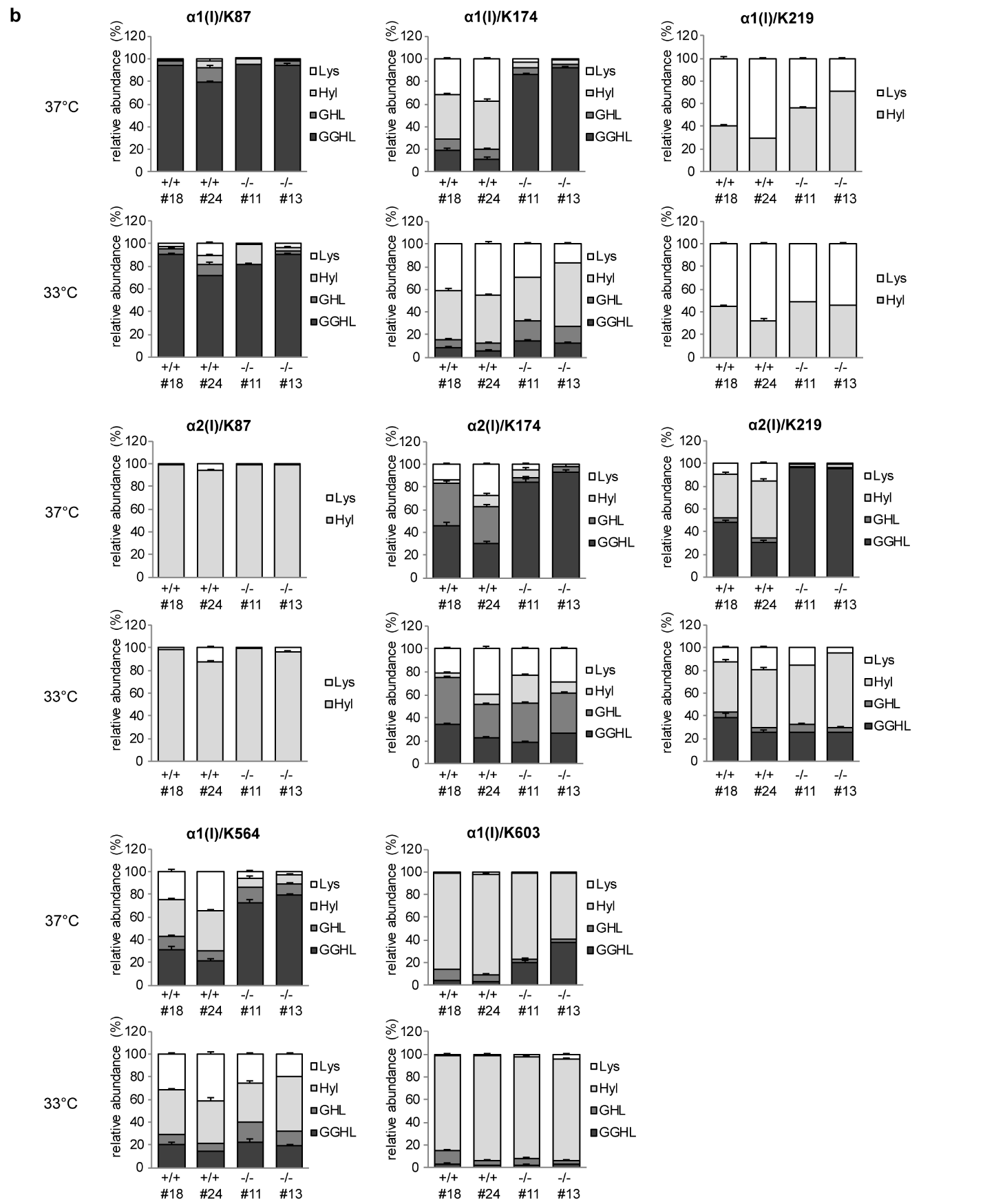


**Figure S1.** *Continued.*


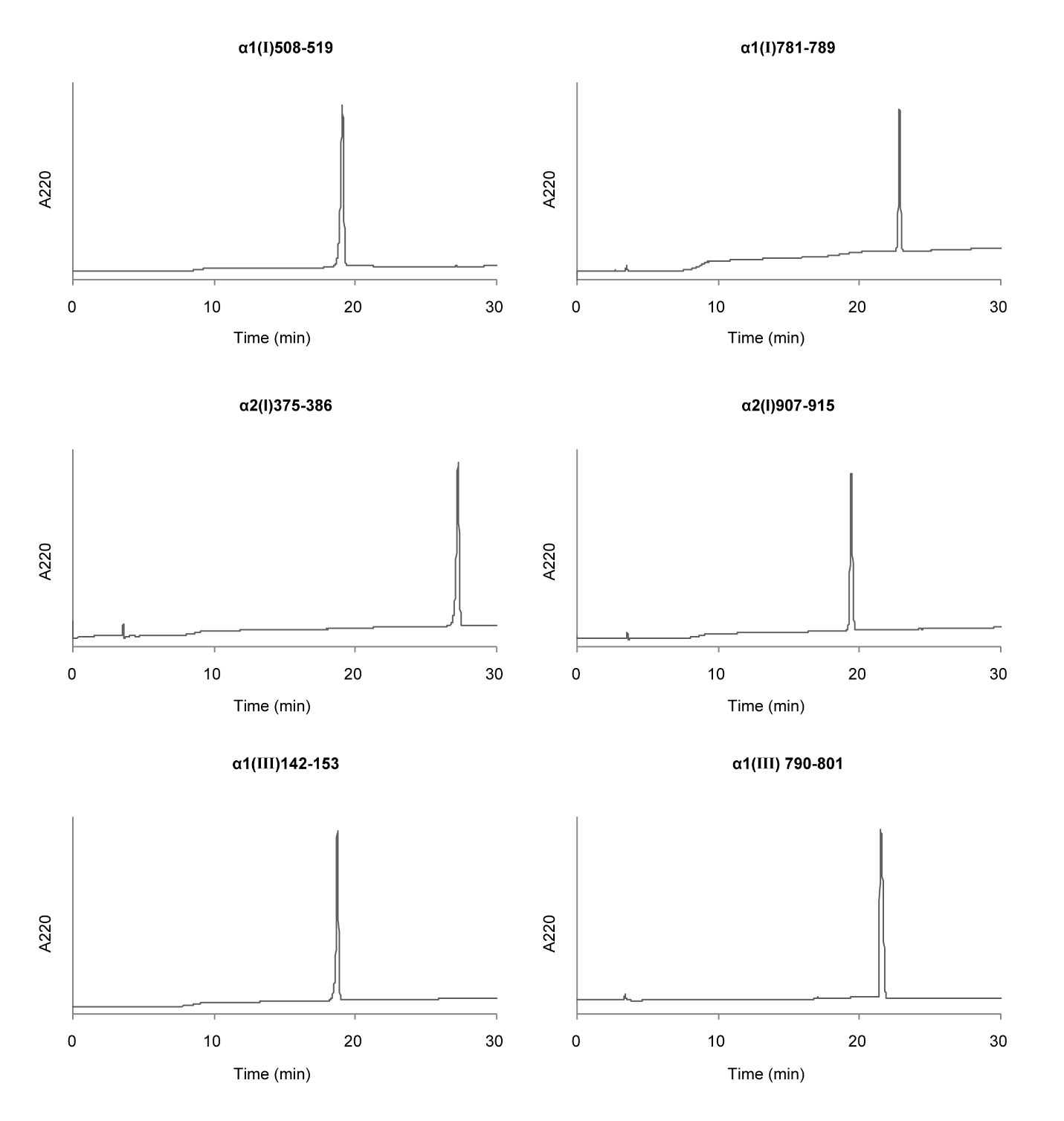


**Figure S2.** **HPLC profiles of the synthesized peptides.**

The purified peptides were analyzed by RP-HPLC using a COSMOSIL 5C_18_-AR-II column (4.6 × 250 mm, Nacalai Tesque). HPLC gradient: 0%–30% CH_3_CN in 0.05% TFA over 30 min. Detection: 220 nm. Flow rate: 1 mL/min.

**Table S1. Quantification of α1(I)-, α2(I)- and α1(III)-chains secreted by MEF clones.**

(a) Collagens secreted by MEF clones were isolated from culture media by salt precipitation after addition of SI-collagen. The collagen sample was heat denatured, followed by trypsin digestion. Tryptic marker peptides for quantification of α1(I)-, α2(I)- and α1(III)-chains were measured by LC-MS. Values are means ± SD (n = 3).

(b) Pepsin-indigestible fractions of α1(I)-, α2(I)- and α1(III)-chains were quantified by LC-MS as described in (a). Values are means ± SD (n = 3).


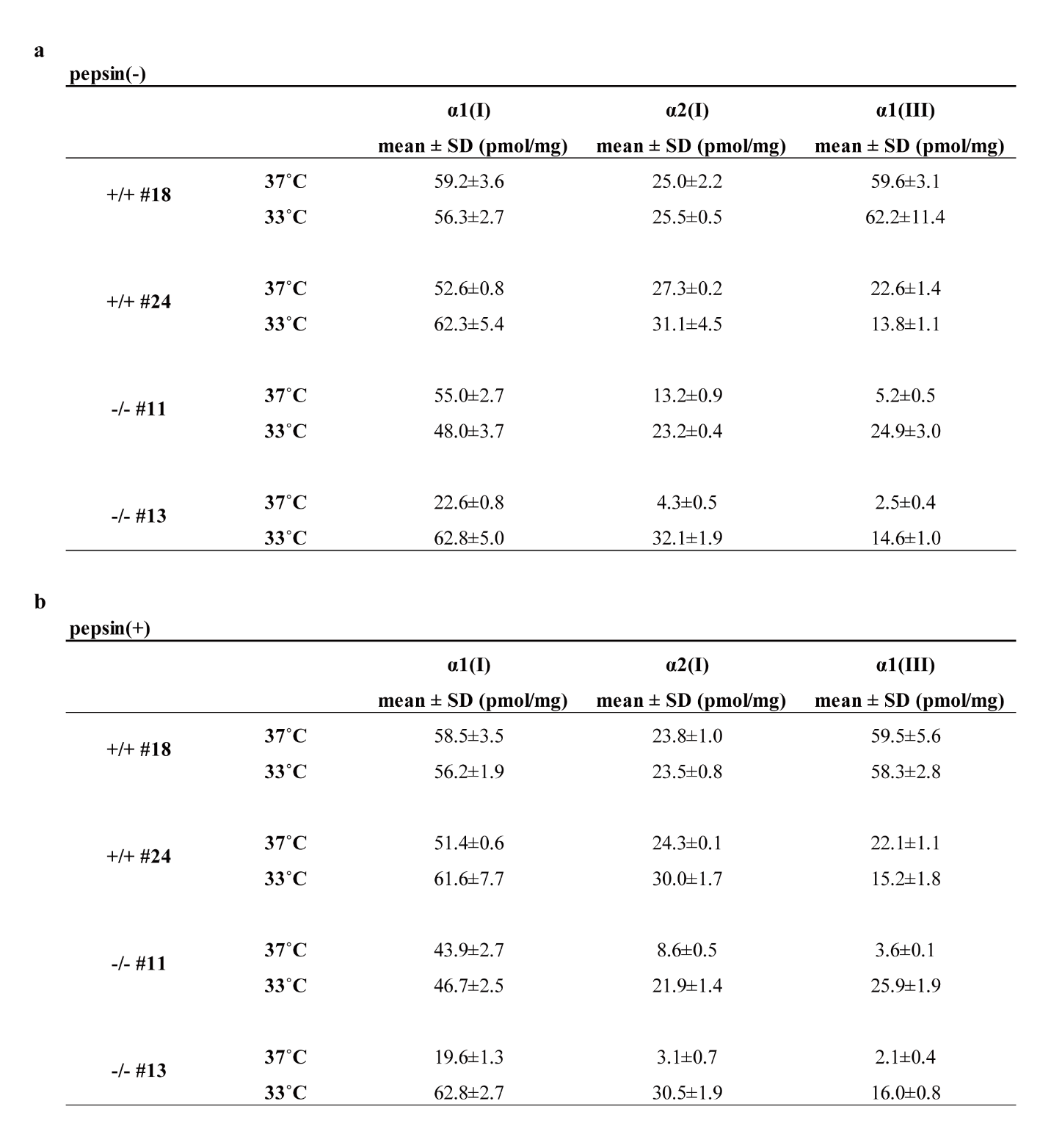


**Table S2.** **Summary of quantitative values of total post-translational modifications in type I collagen shown in Fig. 4.**

Values are means ± SD (n = 3).


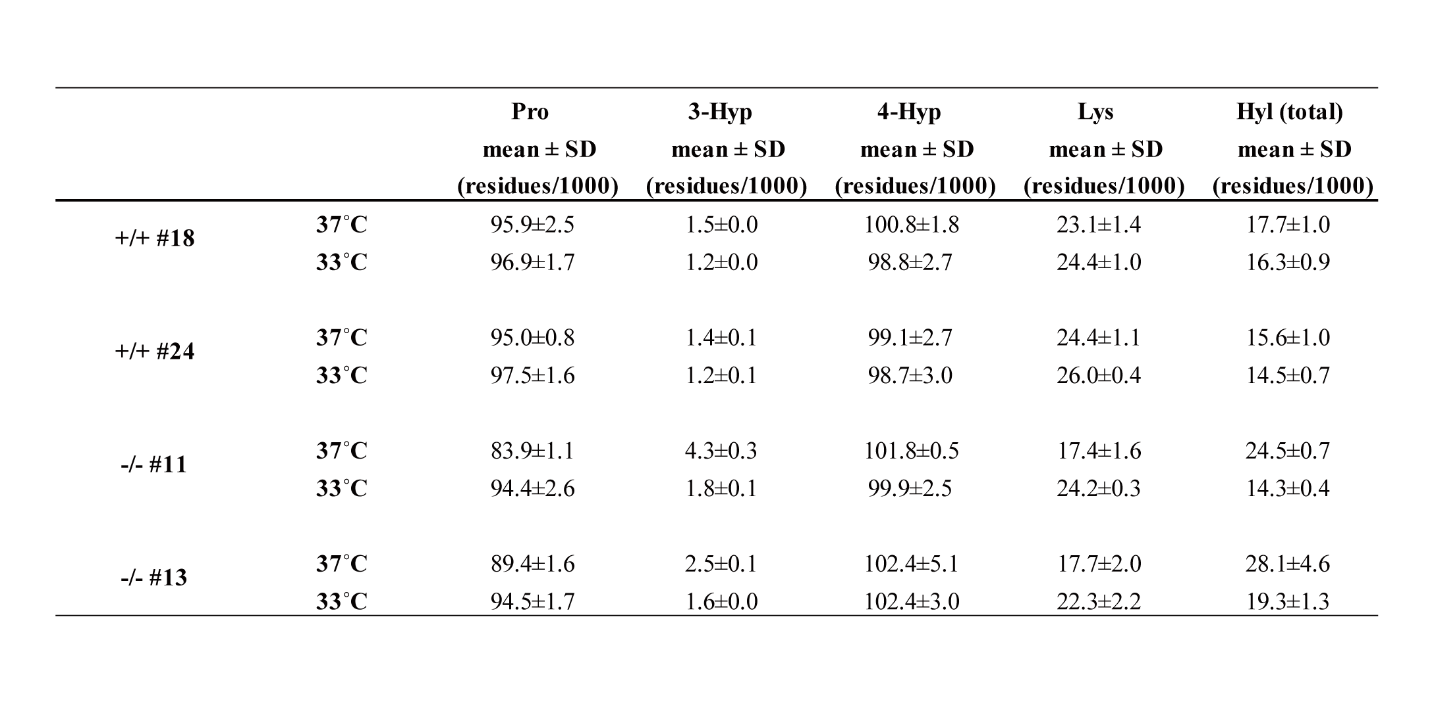


**Table S3. Percentage of prolyl 4-hydroxylation in total Pro residues.**

The percentage of prolyl 4-hydroxylation was calculated based on the quantitative values of Pro, 3-Hyp and 4-Hyp shown in Table S2. Values are means ± SD (n = 3).


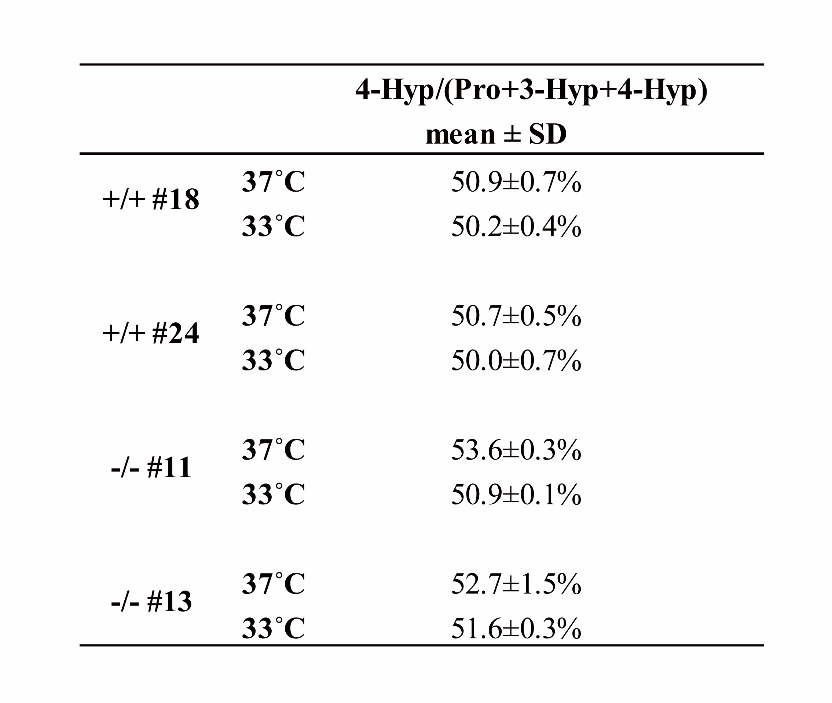


**Table S4.** **Mass spectrometric data of the synthesized peptides.**


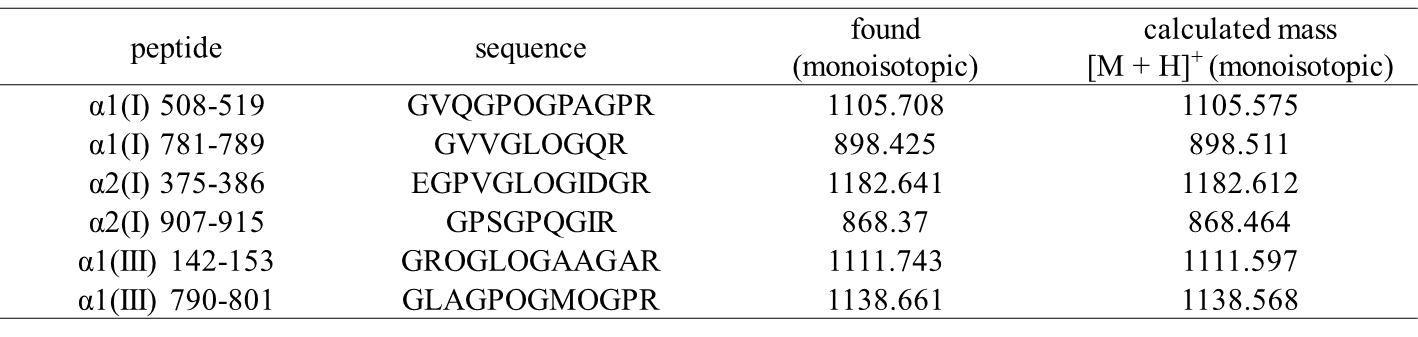


O: 4-hydroxyproline.

**Reference**

1. Terajima, M. *et al.* Cyclophilin B deficiency causes abnormal dentin collagen matrix. *J. Proteome Res.* **16**, 2914–2923 (2017).

**Full-length gels and blots**

Original western blot used in Fig. 1a.

**
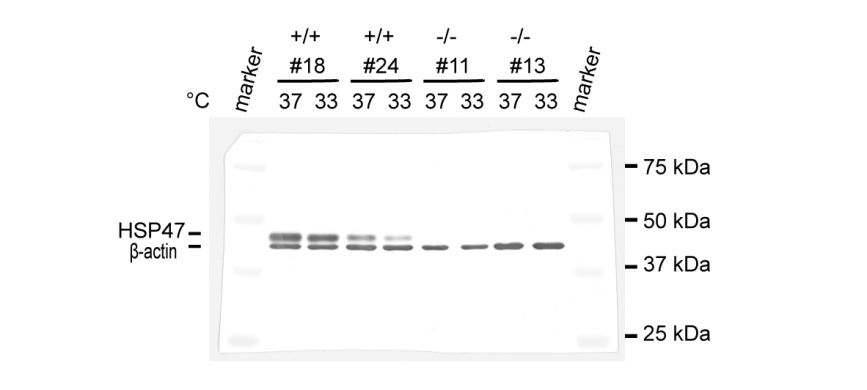
**

Marker: Precision Plus Protein™ All Blue Standards, Bio-Rad

Original western blot used in Fig. 1b.

**
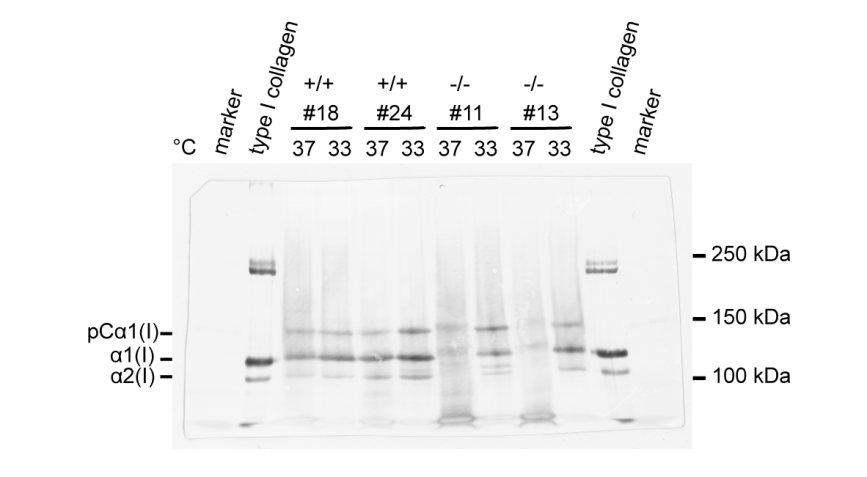
**

Marker: Precision Plus Protein™ All Blue Standards (Bio-Rad)

Type I collagen: IAC-30, Koken

Original western blot used in Fig. 1c.

**
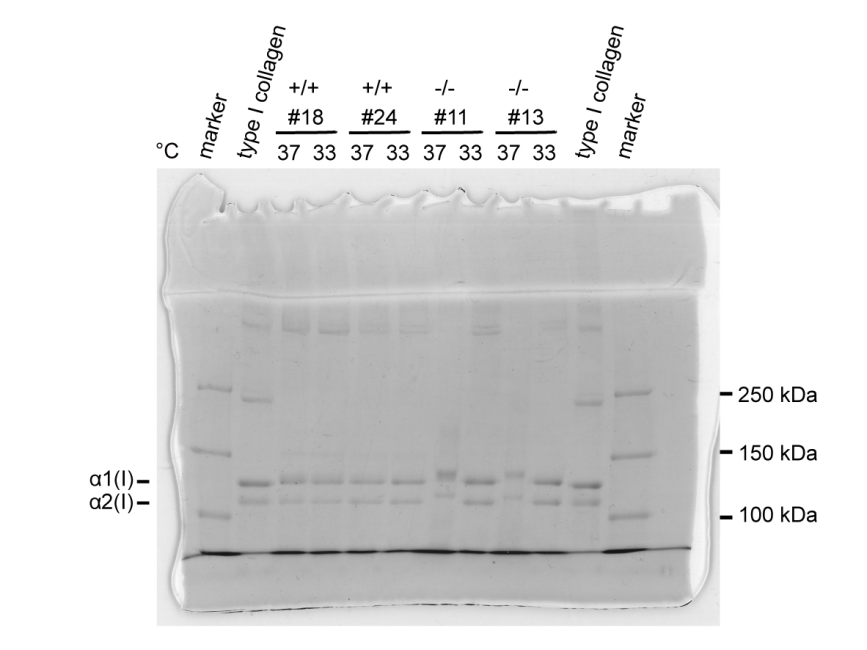
**

Marker: Precision Plus Protein™ All Blue Standards (Bio-Rad)

Type I collagen: IPC-30, Koken

Original western blot used in Fig. 1d.

**
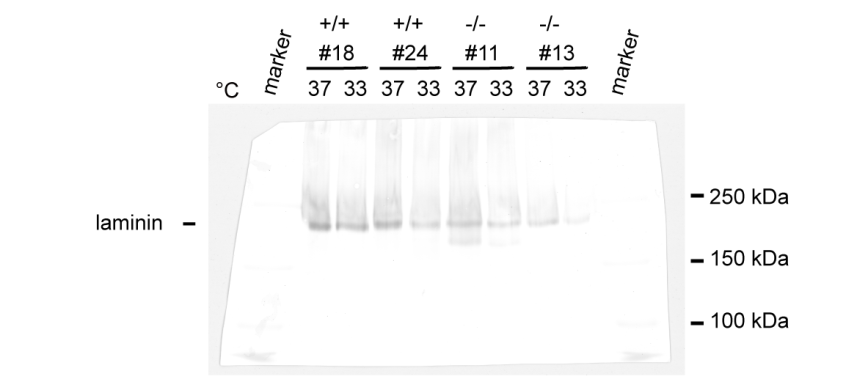
**

Marker: Precision Plus Protein™ All Blue Standards, Bio-Rad

Original western blot used in Fig. 1e.

**
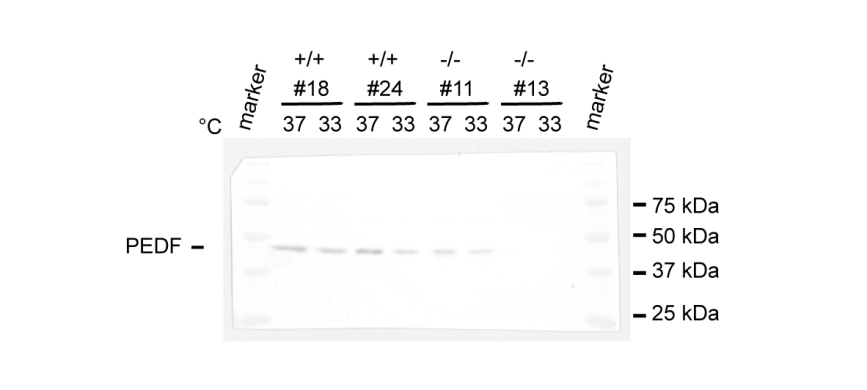
**

Marker: Precision Plus Protein™ All Blue Standards, Bio-Rad

Original gels used in Fig. 5a.






















Marker: Precision Plus Protein™ All Blue Standards, Bio-Rad

Type I collagen: IPC-30, Koken
